# The relationship between naevus count, memory function and telomere length in the Twins UK cohort

**DOI:** 10.1111/pcmr.12722

**Published:** 2018-07-19

**Authors:** Stefano Masi, Georgios Georgiopoulos, Simone Ribero, Stefano Taddei, Veronique Bataille, Claire J. Steves

**Affiliations:** ^1^ Department of Twin Research & Genetic Epidemiology King's College London London UK; ^2^ Department of Clinical and Experimental Medicine University of Pisa Pisa Italy; ^3^ First Department of Cardiology Hippokration Hospital University of Athens Athens Greece; ^4^ Department of Ageing and Health, Guys and St Thomas's NHS Foundation Trust London UK; ^5^ Department of Medical Sciences Dermatologic Clinic University of Turin Turin Italy

**Keywords:** biological ageing, memory decline, naevus count, neurodegeneration, telomere length

## Abstract

The presence of a skin–brain connection whereby alterations in the skin can inform on mechanisms underlying neurodegenerative diseases is increasingly recognized. In this study, we used a discovery (*n* = 321) and replication (*n* = 147) sample from the Twins UK population to test the association between naevus count and memory function, and its mediation by telomeres. Memory function was assessed in 1999 and 2009 using the paired associates learning test (PAL), while naevus count and leucocyte telomere length (LTL, assessed by the terminal restriction fragment assay) were measured once. Higher baseline naevus count was significantly associated with fewer errors at the baseline and follow‐up PAL, as well as with change in PAL score over 10 years. This association was significantly attenuated after adjustment for LTL. The significant association between naevus count and PAL score was reproduced in the replication sample. These findings suggest that melanocytes might be used as model system to study the biological ageing pathways involved in neurodegeneration.

1

**Significance**

The results of our study suggest that shared biological ageing pathways might control melanocyte biology and the process of neurodegeneration leading to memory decline. The limited availability of suitable neuronal models to study the biological alterations underlying neurodegenerative diseases has precluded identification of potential therapeutic targets. Our results suggest that melanocytes and neurons might preserve common biological regulations in postnatal life and support the use of readily available melanocytes from the skin to shed light on the complex biology underlying neurodegenerative diseases.

## 1 INTRODUCTION

The presence of a skin–brain connection, so that alterations detected on the skin might inform on specific neurogenerative processes underlying Parkinson's disease and Alzheimer's disease (AD), is attracting increasing interest (Clos, Kayed, & Lasagna‐Reeves, 2012). Indeed, the biological processes underlying neurodegenerative diseases are complex, and the limited availability of suitable neuronal models for neurodegenerative research has hindered the understanding of the biological pathways involved in the disease initiation and evolution, as well as the identification of potential therapeutic targets.

Several reports have documented deficits of episodic memory in subjects who will develop AD or other neuropsychiatric disorders prior to diagnostically significant cognitive changes. Using data collected within the Twins UK study, which is a volunteer twin registry representative to the broader UK population regarding disease‐related and lifestyle characteristics (Andrew et al., 2001), our group has recently reported an association between short leucocyte telomere length (LTL) and impaired episodic memory (Valdes et al., 2010). This association remained significant after adjustment for chronological age, suggesting that LTL might contribute to characterize the insidious transition from normal ageing to dementia, identifying people within the general population at increased risk of neuropsychiatric disorders because of a faster evolution of biological ageing.

Neurons are long‐living postmitotic cells, which share several features with epidermal melanocytes, including a common embryological origins (Slominski, Tobin, Shibahara, & Wortsman, 2004). Similarly to episodic memory, total body naevus count declines with ageing and we previously documented that shorter LTL is associated with lower number of naevi, even after adjustment for chronological age (Bataille et al., 2007).

This evidence suggests that shared biological ageing pathways, captured by the measure of LTL, might influence both the evolution of neurodegenerative diseases underlying AD and the replicative potential within a naevus clone.

Based on this background, we used two independent samples from the Twins UK population to explore the relationship between naevus count and episodic memory, as well as whether this association could be explained, at least in part, by biological ageing (as assessed by LTL). The first (discovery) sample included 321 participants with measures of episodic memory function available in 1999 and 2009, and whole body naevus count assessed at baseline (Table 1). In this sample, 187 participants also had measures of LTL. The second (replication) sample included 147 participants who underwent at least one memory function and naevus count assessment between 2006 and 2016 (Table 1). Both samples included participants free of overt dementia or cognitive impairment. A naevus was defined as a melanocytic lesion ≥2 mm in diameter to avoid any confusion with ephelides, and the protocol used for whole body naevus counting has been validated in the previous studies (Bataille et al., 1996, 1998). The paired associates learning (PAL) test (adjusted errors at eight boxes), which is part of the computerized CANTAB cognitive test battery (Robbins et al., 1994), was used to assess episodic memory function. This test was selected as previous reports documented its capacity to detect memory changes predictive of AD and to provide a reliable estimate of memory changes in follow‐up studies (Barnett, Blackwell, Sahakian, & Robbins, 2016). Characteristics of the test and the method used for its administration were described in Steves et al. (Steves, Jackson, & Spector, 2013). Mean leucocyte terminal restriction fragment length was used as a measure of LTL and assessed by the Southern blot method, as previously described (Bataille et al., 2007). Ethics Committee approval for the Twins UK study was obtained from the Guy's and St Thomas Hospital NHS Trust, London. Subjects were not aware of the hypotheses being tested in this study at the time of recruitment.

**Table 1:** Clinical characteristics of the study population in the discovery and replication samples

Samples	Variable	*N*	Mean	Std. Dev.
Discovery	Age (years)	276	53	7
	Paired Associates Learning 1999 (total errors)	315	22	20
	Paired Associates Learning 2009 (total errors)	321	22	22
	Leucocyte telomere length (kB pair)	187	7.003	0.581
	Whole body naevus count (*n*)	297	31	38
Replication	Age (years)	145	67	6
	Paired Associates Learning (total error adjusted)	145	17	15
	Whole body naevus count (*n*)	145	36	36

In the discovery sample, change in PAL score for the periods 1999‐2009 conditional on earlier PAL score was calculated by regressing the PAL follow‐up score on the earlier PAL measure, saving and standardizing the residuals. Subsequently, we implemented a series of generalized estimating equations (GEE) to fit population‐averaged panel‐data (i.e., pairs of twins) models with unstructured within‐group correlation for the association of (a) baseline naevus count with memory function both at baseline and follow‐up, (b) baseline naevus count and changes in episodic memory, (c) LTL and naevus count at baseline and d) LTL and episodic memory. Normal distribution of the dependent continuous variables was graphically inspected with histograms and distributional plots (i.e., percentile–percentile plots). Variables that deviated from normality were transformed with the natural logarithm before entering the GEE analysis. Results from the GEE analysis are presented as coefficients (β) and 95% confidence intervals (CI). To explore the proportion of the association between baseline naevus count and change in PAL explained by chronological or biological age, we included age and LTL, separately and in combination, in the models exploring the relationship between baseline naevus count and PAL residuals. The GEE analysis for the association between naevus count and PAL score was repeated in the replication sample. The Stata 13 statistical package was used for all analyses. We deemed statistical significance at *α* = 0.05.

Participants with higher naevus count had a better episodic memory (making fewer errors at the PAL) both at the baseline (*β *= −0.209; 95% CI ‐0.384, ‐0.034; *p* = 0.019) and follow‐up (*β* = −0.401; 95% CI −0.617, −0.186; *p* < 0.001) assessments. Naevus count was also related with LTL, so that people with higher naevus count had longer LTL (*β* = 0.280; 95% CI 0.011, 0.549; *p* = 0.041). In turn, LTL was associated with the performance at the PAL test (*β* = −0.047; 95% CI −0.075, −0.019; *p* < 0.001). All these associations remained significant after adjustment for chronological age. People with higher naevus count at baseline had a lower memory decline during follow‐up (*β* = 0.192; 95% CI 0.075, 0.309; *p* = 0.001) (Table 2). Importantly, while adjustment for age did not affect this association (*β* = 0.216; 95% CI 0.018, 0.414; *p* = 0.032), further adjustment for LTL substantially reduced the strength of the association between naevus count and change in PAL (*β* = 0.095; 95% CI −0.168, 0.358; *p* = 0.478) (Table 3a). In this model, LTL was significantly associated with change in PAL (*β* = 0.495; 95% CI 0.076, 0.914; *p* = 0.020), while chronological age was not (*β* = −0.017; 95% CI −0.049, 0.014; *p* = 0.267).

**Table 2:** Unadjusted associations of naevus count at baseline with (a) paired associates learning test (PAL) results at the same assessment, (b) paired associates learning test (PAL) results at follow‐up, (c) change in paired associates learning test (PAL) and iv) LTL in the discovery sample

	PAL Baseline (1999)		PAL at follow‐up (2009)		Change in PAL		Telomeres (TRF)	
	Coef (95% CI)	*p*	Coef (95% CI)	*p*	Coef (95% CI)	*p*	Coef (95% CI)	*p*
Whole body naevus count	−0.209 (−0.384, −0.034)	0.019	−0.401 (−0.617, −0.186)	<0.001	0.192 (0.075, 0.309)	0.001	0.280 (0.011, 0.549)	0.041

*Note*

Associations were established using generalized estimating equations.

**Table 3:** Unadjusted and multiadjusted association between (a) baseline naevus count and changes in PAL in the discovery sample, and (b) naevus count and PAL test in the replication sample

	Change in PAL					
	Unadjusted		Adjusted for age		Adjusted for age + telomeres (TRF)	
	Coef (95% CI)	*p*	Coef (95% CI)	*p*	Coef (95% CI)	*p*
**(a) Discovery sample**						
Whole body naevus count	0.192 (0.075; 0.309)	0.001	0.216 (0.018; 0.414)	0.032	0.095 (−0.168; 0.358)	0.478
Log (PAL Total Error Adj)						
**(b) Replication sample**	Coef (95% CI)	*p*	Coef (95% CI)	*p*		
Whole body naevus count	−0.003 (−0.006, −0.0005)	0.023	−0.004 (−0.007, −0.001)	0.017		

*Note*

Associations were established using generalized estimating equations.

Following the same statistical approach, but with PAL results log transformed as non‐normally distributed, the association between naevus count and PAL score was confirmed using the cross‐sectional data available in the replication sample (*β *= −0.003; 95% CI −0.006, −0.0005; *p* = 0.023). Similar to the results obtained in the discovery sample, this association was unaffected by adjustment for age (*β *= −0.004; 95% CI −0.007, −0.001; *p* = 0.017) (Table 3b). The limited number of survey participants with telomeres, naevus count and cognitive data in the replication sample did not enable replication of the LTL mediation analysis.

These results show, for the first time, an association between cross‐sectional measures of naevus count and episodic memory, as well as that baseline naevus count can predict decline in episodic memory over 10‐year follow‐up. They also document that these associations are likely to be mediated by pathways regulating biological ageing. Our data support the hypothesis that shared biological ageing pathways influence the process of neurodegeneration associated with decline in episodic memory as well as melanocyte regenerative capacities.

Decline in episodic memory is commonly observed after 30–35 years of age, and its rapid impairment has been linked not only with the risk of AD but also with other neuropsychiatric disorders characterized by reduction in hippocampal volume (Dickerson & Eichenbaum, 2010; Kuhn & Gallinat, 2014; Van Petten, 2004), including bipolar disorder, major depressive disorder and schizophrenia (Bora, Harrison, Yucel, & Pantelis, 2013; Bora, Yucel, & Pantelis, 2009; Bourne et al., 2013; Lim et al., 2013; Szoke et al., 2008). As telomere length controls cellular replication capacities and pathways of cellular senescence/degeneration, an altered telomere dynamic might account for a faster hippocampal volume loss which, in turn, might represent the morphological substrate accounting for the faster impairment in episodic memory observed in subjects with shorter LTL in this and previous studies (Valdes et al., 2010). In keeping with this hypothesis, several reports described associations between hippocampal volume and LTL (King et al., 2014; Nilsonne, Tamm, Mansson, Akerstedt, & Lekander, 2015). Like episodic memory, loss and degeneration of skin melanocytes determine a progressive decline in naevus count from the third decade of life and we previously documented that a lower total body naevus count is associated with shorter LTL (Bataille et al., 2007). Taken together, this evidence suggests that the rate of decline in naevus count might help identification of people within the general population with altered regulation of biological ageing pathways, thus with a greater risk of hippocampal degeneration, episodic memory decline and, ultimately, neurodegenerative diseases. This is confirmed by genetic studies showing that single nucleotide variants or epigenetic modifications in the region of TERT are associated with naevus count (Roos et al., 2017) and the risk of Alzheimer's disease (Zhan et al., 2015).

Our study has several strengths. The availability of robust memory measures assessed at two different time points 10 years apart reduces the risk of learning effects, often complicating the interpretation of changes in cognitive function observed in longitudinal studies. Previous reports have validated the PAL tests for the assessment of episodic memory deficits, showing its ability to detect early memory changes predictive of AD risk. LTL was measured using terminal restriction fragment, which is currently considered the gold standard method for LTL assay. Finally, the work previously published by our group on the relationship of LTL with episodic memory as well as with naevus count increases the robustness of our results. Among limitations, while the association between naevus count and memory function was replicated in two independent samples, the size of both samples was relatively small. Also, while several reports have now demonstrated that telomere length is highly synchronized between different cells and tissues at any age (Butler et al., 1998), telomeres were measured in peripheral leucocytes and not directly on melanocytes and/or neurons.

In conclusion, the inverse association between naevus count and risk of episodic memory decline observed in our study suggests that a faster age‐related decline in naevus count might help identification of people with faster evolution of biological ageing, and thus at greater risk of neuropsychiatric disorders. Also, our findings suggest that peripheral melanocytes could maintain common biological regulation with neurons in postnatal life, at least for pathways influencing the progression of biological ageing. Consequently, clarification of the mechanisms involved in the control of biological ageing of peripheral melanocytes might provide valuable information on the potential pathways involved in neurodegeneration and leading to memory decline.
